# Khat chewing prevalence and correlates among university staff in Ethiopia: a cross-sectional study

**DOI:** 10.1186/s13104-019-4706-1

**Published:** 2019-10-21

**Authors:** Yigizie Yeshaw, Muluken Fekadie Zerihun

**Affiliations:** 1 Department of Medical Physiology, School of Medicine, College of Medicine and Health Science, University of Gondar, Gondar, Ethiopia; 2 Department of Biochemistry, College of Medicine and Health Sciences, Gondar University, Gondar, Ethiopia

**Keywords:** Khat chewing, Ethiopia, University staff, Associated factors

## Abstract

**Objectives:**

Khat is an herbal cultivated plant growing mainly in Eastern Africa and Arabians. Chronic khat chewing leads to the development of liver toxicity, cardiovascular disease, sleeping disorder, psychosis, memory impairment, poor academic performance and variety of social and economic problems affecting the consumers and their families. Therefore, this study aimed to determine the prevalence of khat chewing and associated factors among Jimma university staff. An institution-based cross-sectional study was employed on 354 university staff. A self-administered questionnaire was used to collect the data. The analysis was done using Stata 14. A multivariable logistic regression model was used to identify associated factors of khat chewing among university staff. p ≤ 0.05 was employed to declare statistically significant factors.

**Results:**

The lifetime prevalence of khat chewing among university staff was 41.0% (95% CI 35.9–46.1%). The odds of khat chewing was higher among males (AOR = 2.66 95%, CI 1.52–4.68), participants who had khat chewer friends (AOR = 2.15 95% CI 1.18–3.93), alcohol users (AOR = 9.02, 95% CI 4.96–16.42) and participants who had khat chewer family member (AOR = 4.03, 95% CI 1.16–13.99). Since a significant proportion of staff chew khat, appropriate measures need to be taken to reduce its prevalence and adverse social and health consequences.

## Introduction

Khat (Catha edulis) is an herbal trade-related cultivated plant growing in most parts of the world particularly in Eastern Africa and Arabians [1–3]. It contains psychoactive substances cathinone and cathine, which can cross the blood–brain barrier to enter and stimulate the brain [1, 2, 4, 5]. The major constitute of khat, cathinone is a sympathomimetic drug, works through stimulating the autonomic sympathetic nervous system, commonly known as the ‘fear-flight’ response [6].

In Ethiopia, khat chewing has a deep-rooted history and is commonly used for social and religious purposes [2]. According to the report of 2016 Ethiopian Demographic Health Survey, 12% of women and 27% of men have ever chewed chat [7]. The prevalence of khat chewing was 46% among university staff of Jimma University [8], 7% among medical students of Addis Ababa University [9], 16% and 37.8% among high school students of Jimma city [10], 16.3% among Hawassa University students [11], 13.6% among Gondar university students [12], 40.0% among Adama university students [13], and 6.67% among Adigrat University students [14].

Associated factors of khat chewing include: being male [10, 15–17], urban in residence [15], age group between 19 and 23 years [10], peer influence [11, 12, 16], family history of chewing khat [18], having khat chewer friends [19], richest wealth quintiles [17] and alcohol drinking [16, 19]. Cigarette smoking, increased workload, and religious practice were also found to be independent predictors of chewing [12].

The habit of khat chewing lead to both acute and chronic effects like low birth weight, reduced sperm count and motility, social and economic problems affecting the consumers and their families [5]. It is also associated with liver toxicity [4, 20, 21], cardiovascular disease [2, 4, 21, 22], periodontal disease [2, 21, 23], sleeping disorder [24], reduced appetite [4, 24], depression [4, 24–26] gastrointestinal adverse effect [2, 4, 21, 24], psychosis [2, 4, 21], memory impairment [4] and poor academic performance [25, 27].

In spite of the above problems of khat chewing, a significant number of staff in higher education institutions chew khat to “increase” their concentration levels and attention span [28] and spent long hours to chewing and then recovering from chewing; this causes absenteeism from work, poor academic performance and under employment [29, 30].

Therefore, this study will give insight to policymakers and next researchers in the area by identifying the prevalence of khat chewing and associated factors among university staff, which is not well investigated so far.

## Main text

### Methods

#### Study area and period

An institution-based cross-sectional study was conducted at Jimma University College of Health Sciences from March 24 to April 24, 2016. There are a total of 3395 staff at Jimma University College of Health Sciences and Jimma University Hospital staff.

#### Source and study population

All academic and administrative staff of the College of Health Sciences and Jimma University Hospital were the source population. All academic and administrative staff of the College of Health Sciences and Jimma university hospital who were present during the data collection period were the study population.

#### Inclusion and exclusion criteria

Participants who were severely ill and having hearing and speaking difficulty were excluded.

#### Sample size and sampling procedure

The sample size for this study was calculated using single population proportion formula, by considering 46% proportion of khat chewing in another study [8], 5% margin of error and 95% confidence interval. Accordingly, the calculated sample size for the study was 381. Since the source population was 3395 (< 10,000), a finite proportion correction formula was used, which gives a sample size of 343. After adding a non-response rate of 5%, the final sample size of the study was 360. After taking the list of university staff from the human resource office of Jimma University, computer-generated simple random sampling technique was employed to select the study participants for this study.

#### Data collection procedure

The data were collected by four psychiatry nurses using pretested self-administered questionnaire and training was given for data collectors before the data collection period.

#### Operational definitions

Lifetime khat chewer is an individual who had ever used khat at least once in his/her lifetime.

Current khat chewer is an individual who had used khat at least once in the last month before the study.

Lifetime cigarette smoker is an individual who had ever used cigarette at least once in his/her lifetime.

Lifetime alcohol drinker is an individual who had ever used alcohol drinks such as traditional alcohol drinks of Ethiopia (such as tela, tej, katicala/areki), beer, wine, or other drinks that can cause intoxication at least once in his/her lifetime.

#### Data quality control

To assure data quality first, the questionnaire was pre-tested and training was given for data collectors. Data were also appropriately entered and coded before analysis.

#### Data processing and analysis

Data were checked for completeness and entered into Epi-data version 3.1. Then, it was exported to Stata 14 software for further analysis. Bivariable logistic regression analysis was performed to find the association of each independent variable with khat chewing. All variables with a *p* value of 0.25 at bivariable logistic regression analysis were entered into the multivariable logistic regression model. p ≤ 0.05 was considered statistically significant. Adjusted odds ratio (AOR) and its 95% confidence interval (CI) was calculated for potential associated factors included in the final model.

### Results

#### Socio-demographic and substance use-related characteristics of respondents

Three hundred and sixty subjects were included in the study and the overall response rate was 354 (98.3%). More than half (52%) of respondents were females. One hundred and fifty-five (43.8%) respondents were Orthodox Christians and followed by Muslims 110 (31.1%). The majority, 194 (54.8%) of respondents were Oromo. One hundred and sixty-six of them (46.9%) were married. One-third of the respondents (33%) had khat chewing friend and 159 (44.9%) of respondents were not satisfied with their current job (profession). Regarding substance use, 205 (57.9%) and 17 (4.8%) of respondents had used alcohol and cigarette respectively (Table 1).

**Table 1:** Socio-demographic and substance use-related characteristics of Jimma university staff, Jimma, Ethiopia, 2016

Variables	Frequency	Percent
Sex		
Female	165	52.3
Male	169	47.7
Religion		
Orthodox	155	43.8
Muslim	110	31.1
Protestant	80	22.6
Catholic	9	2.5
Ethnicity		
Oromo	194	54.8
Amhara	85	24
Gurage	36	10.2
Tigray	29	8.2
Other^a^	10	2.8
Age (years)		
18–24	71	20.1
25–34	223	63.0
35–44	35	9.9
≥ 45	25	7.1
Marital status		
Married	166	46.9
Single	158	44.6
Divorced	19	5.4
Widowed	11	3.1
Educational status		
Secondary	37	10.5
Diploma	143	40.4
Degree	146	41.2
Masters and above	28	7.9
Salary (ETB)		
550–1114	94	26.6
1115–1800	88	24.9
1801–3144	65	18.4
≥ 3145	107	30.2
Had khat chewer friends		
No	237	67
Yes	117	33.0
Job satisfaction		
No	159	44.9
Yes	195	55.1
Alcohol use		
No	149	42.1
Yes	205	57.9
Cigarrete smoking		
No	337	95.2
Yes	17	4.8
Family history of chewing khat		
No	330	93.2
Yes	24	6.8

^a^Wolayita, Kefa, and Dawero

#### Prevalence of khat chewing and its associated factors

The lifetime prevalence of khat chewing among university staff was 41% (95% CI 35.9–46.1%). Of these, 102 (70.3%) of them chew khat every day, 25 (17.2%) of them chew khat 2 to 3 times per week and the rest 18 (12.4%) chew khat once a week. Of the total 145 khat chewers, 97 (66.9%) of them were current khat chewers. On bivariable logistic regression analysis: sex, age, marital status, education level, job satisfaction, alcohol use, having a family history of khat chewing and khat chewing friends were associated with khat chewing (p < 0.25). However, in the final model: only sex, alcohol use, having a family history of khat chewing and khat chewing friends were significantly associated with khat chewing practice among university staff (p ≤ 0.05).

Males had 2.66 times higher odds to chew khat than females (AOR = 2.66 95% CI 1.52–4.68). The odds of khat chewing among participants who had khat chewer friends was 2.15 times higher compared to participants who didn’t have khat chewer friends (AOR = 2.15 95% CI 1.18–3.93). The odds of khat chewing among alcohol drinker participants was 9 times higher compared to non-drinkers (AOR = 9.02, 95% CI 4.96–16.42). The odds of khat chewing among participants with a family history of khat chewing was 4 times higher compared to those staff who didn’t have (AOR = 4.03, 95% CI 1.16–13.99) (Table 2).

**Table 2:** Bivariable and multivariable logistic regression analysis for the prevalence of khat chewing among Jimma university staff, Jimma, Ethiopia, 2016

Variables	Khat chewing		Odds ratio	
	Yes	No	COR	AOR
	N (%)	N (%)	(95% CI)	(95% CI)
Sex				
Male	87 (51.5)	82 (48.5)	2.32 (1.51–3.58)	2.66 (1.52–4.68)*
Female	58 (31.4)	127 (68.6)	1	1
Age in years				
18–24	16 (22.5)	55 (77.5)	4.38 (1.66–11.50)	1.41 (0.38–5.30)
25–34	96 (43.1)	127 (56.9)	1.68 (0.73–3.87)	0.76 (0.24–2.44)
35–44	19 (54.3)	16 (45.7)	1.07 (0.38–3.01)	1.03 (0.26–4.04)
≥ 45	56 (56.0)	11 (44.0)	1	1
Marital status				
Married	68 (41.0)	98 (59.0)	1	1
Single	57 (36.1)	101 (63.9)	1.23 (0.79–1.93)	1.74 (0.97–3.14)
Divorced	11 (57.9)	8 (42.1)	0.50 (0.19–1.32)	0.82 (0.24–2.80)
Widowed	9 (81.8)	2 (18.2)	0.115 (0.03–0.74)	0.34 (0.05–2.29)
Education level				
Secondary	11 (29.7)	26 (70.3)	3.15 (1.13–8.81)	1.14 (0.33–4.00)
Diploma	57 (39.9)	86 (60.1)	2.01 (0.89–4.57)	0.64 (0.23–1.78)
Degree	61 (41.8)	85 (58.2)	1.86 (0.82–4.21)	0.79 (0.29–2.19)
Masters and above	16 (57.1)	12 (42.9)	1	1
Job satisfaction				
Yes	71 (36.4)	124 (63.6)	1	1
No	74 (46.5)	85 (53.5)	1.52 (0.99–2.33)	0.79 (0.44–1.41)
Had khat chewing friends				
Yes	66 (56.4)	51 (43.6)	1	1
No	79 (33.3)	158 (66.67)	0.39 (0.25–0.61)	2.15 (1.18–3.93)*
Alcohol use				
Yes	124 (60.5)	81 (39.5)	1	1
No	21 (14.1)	128 (85.9)	9.33 (5.44–16.01)	9.02 (4.96–16.42)*
Family history of khat chewing				
Yes	20 (83.3)	4 (16.7)	1	1
No	125 (37.9)	205 (62.11)	8.2 (2.74–24.54)	4.03 (1.16–13.99)*

* p ≤ 0.05

### Discussion

The finding of this study showed that a significant proportion of university staff chew khat, which requires great attention of university managers and other stakeholders. Sex, alcohol drinking, having khat chewer friends and khat chewer family members were associated with khat chewing practice among university staff.

The prevalence of khat chewing in this study was 41%. This finding was in line with the finding of studies conducted among students of Jimma city (37.8%) [31], Adama University (40.0%) [13] and staff of Jimma University (46%) [8]. However, the current finding is higher than the finding of studies conducted among students of Addis Ababa University (7%) [9], Jimma city (16%) [10], Hawassa (16.3%) [11] and Gondar University (13.6%) [12]. The consistency of this finding and the first mentioned three studies might be due to the similarity in study setting (they were conducted in Oromia region), a region where khat is mostly grown and a deep-rooted cultural practice as well [16, 28]. However, as stated in the above statement, the finding of this study had great discrepancy with the result of the last four studies. This could be due to study population differences, in which the current study was conducted among university staff, part of the population with higher income to buy what they want including khat compared to students. Moreover, the presence of strong rules and regulations in the university that prohibit students from using khat and other substances might lower the prevalence of khat chewing practice among students compared to university staff.

Males had 2.66 times higher odds to chew khat than females. This finding is in agreement with the finding of many other studies [10, 15–17]. This similarity could be due to the common trend of social and cultural restrictions to females towards khat chewing practice compared to males in Ethiopia [32].

The odds of khat chewing among alcohol drinker participants was 9 times higher compared to non-drinkers. This is consistent with the finding of other studies in Ethiopia [16, 19]. This could be because most of the time alcohol drinkers are forced to use other psychoactive substance like khat and cigarette.

The odds of khat chewing among participants who had khat chewer friends was 2.15 times higher than participants who didn’t have khat chewer friends. This is consistent with the finding of another study [19]. This is could be due to youth directly persuade their friends to conform to their behavior; therefore khat chewers encourage their inexperienced peers to chew khat [33].

The odds of khat chewing among participants with a family history of khat chewing was 4 times higher compared to those staff who didn’t have. This finding is in agreement with the finding of another study in Ethiopia [18]. This is because respondents who had a family history of chewing tend to imitate and exercise what they observe from their family members.

## Limitation of the study

This study is not free from limitation. Since the issue is sensitive, social desirability bias may be there. The cross-sectional nature of the study design may not allow establishing cause-and-effect relationships among the variables. Thus, it is not possible to identify whether khat chewing influences the associated factors or vice versa. Besides, the above limitation, we tried to identify the magnitude and the possible associated factors of khat chewing using probability sampling method.

## Data Availability

All data underlying the findings are fully available without restriction. All relevant data are within the manuscript.

## Abbreviations

AOR: adjusted odds ratio
CI: confidence interval
